# A New NOTCH3 Gene Mutation Associated With a CADASIL (Cerebral Autosomal Dominant Arteriopathy With Subcortical Infarcts and Leukoencephalopathy) Diagnosis

**DOI:** 10.7759/cureus.26495

**Published:** 2022-07-01

**Authors:** Daniela Neto, Marta Cunha, Filipe Gonçalves, Jorge Cotter

**Affiliations:** 1 Internal Medicine, Hospital da Senhora Da Oliveira, Guimarães, PRT; 2 Internal Medicine, Hospital da Senhora da Oliveira, Guimarães, PRT

**Keywords:** genetic, stroke, paresthesias, notch3, subcortical ischemic leukoencephalopathy (cadasil)

## Abstract

Cerebral autosomal dominant arteriopathy with subcortical infarcts and leukoencephalopathy (CADASIL) is the most common and best-known monogenic small vessel disease. This disease is caused by a genetic mutation in the neurogenic locus notch homolog protein 3 (NOTCH3) gene, inherited as an autosomal dominant trait, the presence of which confirms the diagnosis of CADASIL. Clinically, it can express itself in a variety of symptoms, including migraine with aura, mood disturbance, vascular dementia, ischemic stroke, and premature death. This case reports a 69-year-old man who was admitted for an etiological study of paresthesias and was later confirmed with a diagnosis of CADASIL with a NOTCH3 mutation.

## Introduction

Cerebral autosomal dominant arteriopathy with subcortical infarcts and leukoencephalopathy (CADASIL) is the most common inherited cause of stroke and vascular dementia in adults caused by a genetic mutation in the neurogenic locus notch homolog protein 3 (NOTCH3) gene. It usually manifests itself in middle-aged individuals and can present as a wide variety of symptoms [1]. Other symptoms, in addition to vascular dementia and stroke, include mood disturbances, cognitive dysfunction, and migraine [2]. CADASIL is a rare diagnosis [3]. It is still frequently underdiagnosed, and its minimum prevalence is estimated to be between 2 and 5 in 100,000 and may vary between populations [4].

In order to make a diagnosis of CADASIL, in addition to the symptoms presented, brain MRI findings are of special importance, and confirmation is made through the identification of the NOTCH3 gene mutation [5]. Mutation of the NOTCH3 gene on the short arm of chromosome 19 is the hallmark of CADASIL [4]. This mutation is responsible for recurrent subcortical ischemic infarcts, as it produces thickening and fibrousness of small and medium caliber arteries [6] with obstruction of blood flow [4]. More than 200 different NOTCH3 gene mutations associated with CADASIL have been reported [4]. There is no curative treatment described for CADASIL. Given the evidence of a more severe disease course in individuals with vascular risk factors, control of vascular risk factors is an important part of CADASIL management [4].

This case concerns a 69-year-old male patient with a described history of schizophrenia who presented with paresthesias of the right upper limb. Until the final diagnosis, several possible etiologies for the presented symptoms were equated. He was eventually diagnosed with CADASIL after a compatible brain MRI scan and identification of a mutation in the NOTCH3 gene, although a mutation is not yet described on any population basis.

## Case presentation

A 69-year-old male patient presented to the emergency department with paresthesia in the right upper limb for about two days without other associated symptoms. As a relevant personal history, he had cardiovascular risk factors (hypertension, non-insulin-treated type 2 diabetes mellitus, dyslipidemia), schizophrenia, benign prostatic hyperplasia, smoking habits (60 UMAs), and alcoholic habits (consumption of 50 g of alcohol/day).

He was chronically medicated with ramipril 10 mg once a day, lercanidipine 10 mg once a day, acetylsalicylic acid 150 mg once a day, pitavastatin 2 mg once a day, finasteride 5 mg once a day, silodosin 8 mg once a day, and aripiprazole 10 mg twice a day.

On objective examination, he had a Glasgow Coma Scale of 15/15 (eye (E) 4, verbal (V) 5, motor (M) 6), and a blood pressure of 201/99 mmHg, his heart rate was 71 beats/minute, and he was listless. His neurological examination showed normal mentation with intact cranial nerves, with unremarkable findings from his funduscopic examination, no speech changes (notably no dysarthria or aphasia), he had normal volume in all muscles, no fasciculations or tremors, his muscle strength was globally preserved (tested on the Motor Research Council scale with 5/5 in all muscle groups), osteotendinous reflexes were normal, the cutaneous-plantar reflex was in bilateral flexion, and he had no changes in ambulation or sensory changes.

On suspicion of stroke, a cranial computed tomography (CT) scan was performed, ruling out acute ischemic or hemorrhagic lesions, and an electrocardiogram revealed previously unknown atrial fibrillation (AF).

The patient was admitted for the remaining etiologic study. During the entire hospital stay, different tests were performed, such as blood tests that showed no relevant changes, namely, the autoimmunity and thrombophilia study were negative.

The carotid duplex showed no hemodynamically significant stenosis bilaterally. Transthoracic echocardiogram showed mild left atrial dilatation, moderate eccentric left ventricular hypertrophy, and no other changes.

**Figure 1:**
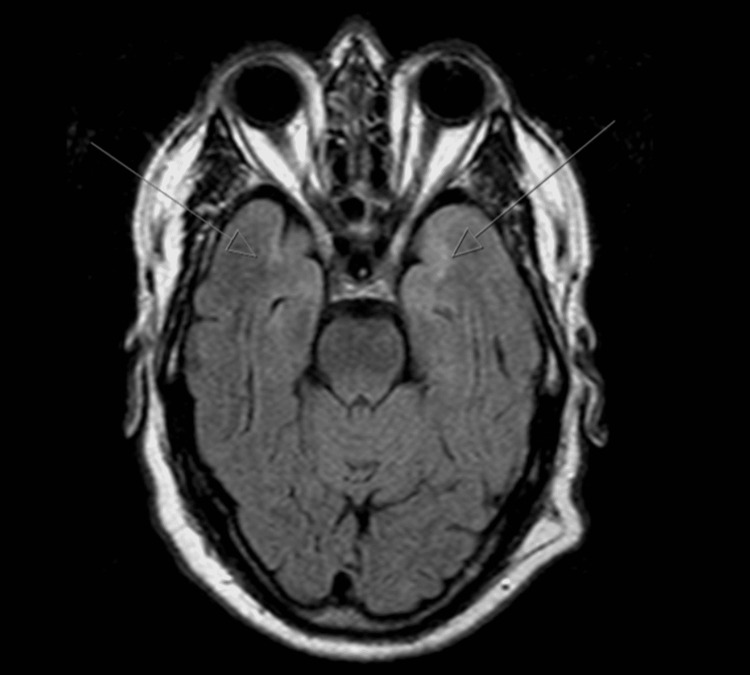
Brain MRI axial FLAIR showing hyperintensities in the anterior temporal lobe bilaterally FLAIR: fluid-attenuated inversion recovery

**Figure 2:**
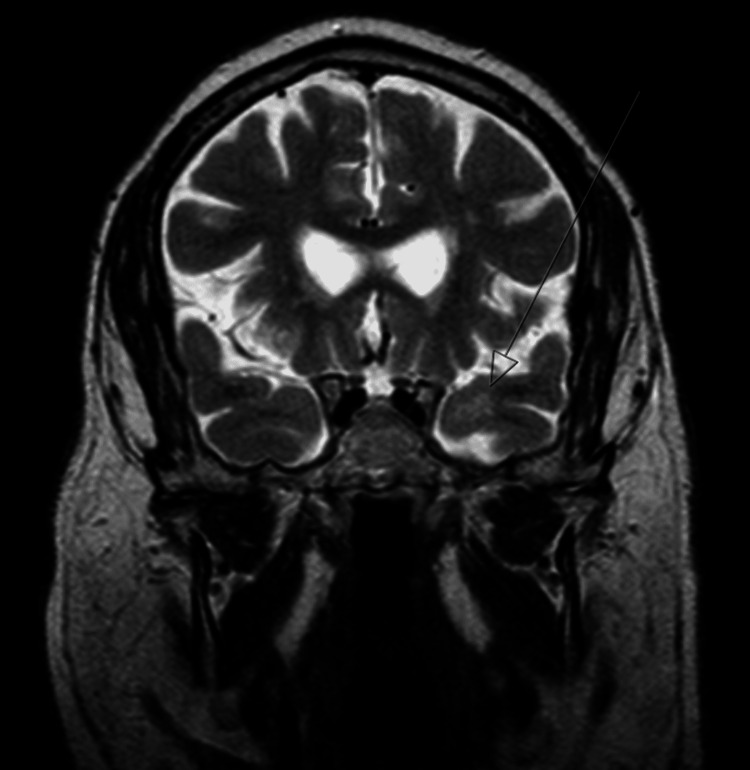
Brain MRI coronal T2 showing hyperintensity in the left anterior temporal lobe

**Figure 3:**
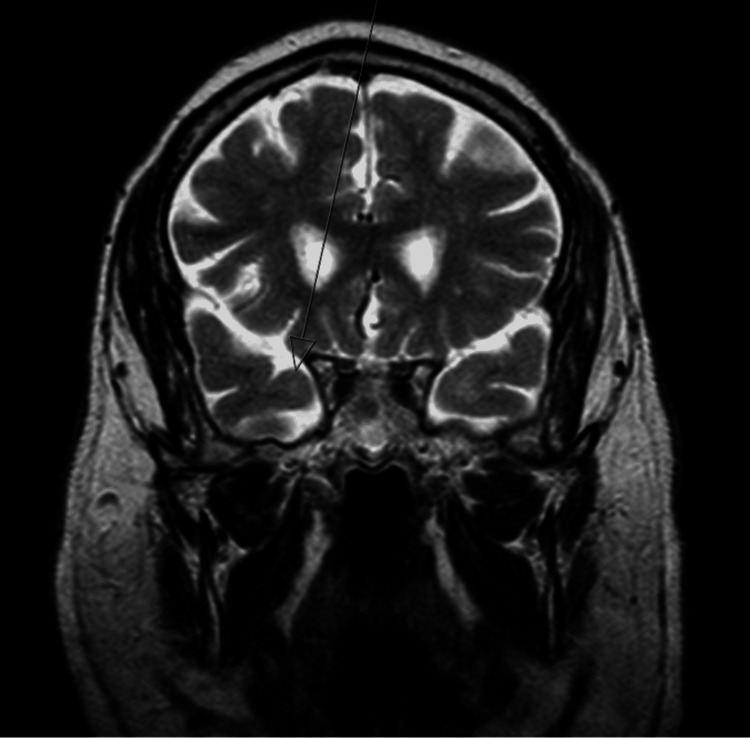
Brain MRI coronal T2 showing hyperintensity in the right anterior temporal lobe

Brain MRI demonstrated foci of hypersignal on TR-long weightings, involving the periventricular white matter (namely, adjacent to the frontal horns and trigones), subcortical frontoparietal, hypersignal being also observed in both temporal poles (left predominant) (Figures 1-3), without restriction to water molecules diffusion, corresponding to probable gliotic-ischemic foci, with associated ischemic lacunae, of a non-recent character, in the right thalamus and left lenticulocapsular. Given the topography of the lesional areas and the clinical context, the CADASIL hypothesis was considered.

During hospitalization, the patient had a controlled tension profile with amlodipine 10 mg once a day, ramipril 10 mg once a day, and a hyposaline diet. He maintained paresthesias of the right upper limb, and behavioral changes with delusional self-referential and persecutory ideas, which motivated therapeutic changes, including dose adjustment of aripiprazole and introduction of donepezil. Given the suspicion of CADASIL, a genetic study was performed.

The patient was clinically discharged and referred to internal medicine and psychiatry outpatient consultation. At discharge, he maintained the medication taken during hospitalization, hypocoagulation for AF, statin, antihypertensive therapy, and psychotropic drugs.

The genetic study detected an “exon 28 DUP variant,” in heterozygosity, in the NOTCH3 gene, which implies the duplication of exon 28 of the NOTCH3 gene (no information is available about its pathogenicity or clinical significance and it is not represented in the population databases consulted, so its frequency in controls is expected to be very low).

After compatible brain MRI imaging and the genetic test with a mutation in the NOTCH3 gene, CADASIL was diagnosed. In the outpatient follow-up, the patient remained asymptomatic. The family was advised to undergo a genetic study.

## Discussion

This case portrays a patient with a diagnosed history of schizophrenia, cardiovascular risk factors (hypertension, non-insulin-treated type 2 diabetes mellitus, dyslipidemia), and smoking habits who was admitted to study the etiology of paresthesias in the right upper limb. Initially, it was considered to be a stroke, but with the clinical correlation with brain magnetic resonance images and later with the identification of the NOTCH3 gene mutation, CADASIL was diagnosed. The diagnosis of CADASIL is based on the combination of clinical and imaging findings on brain MRI and confirmed by genetic analysis. However, it is a major medical challenge due to its rarity [7].

CADASIL can present through a variety of key symptoms such as migraine, cerebral ischemic events, psychiatric problems, including apathy and mood disturbances, and cognitive problems, including dementia. There is great variability in the presentation and progression of symptoms, which is caused by environmental factors such as smoking but also by the presence of vascular disease [8].

The NOTCH 3 gene mutation is 100% sensitive and specific in cases of CADASIL [5]. This gene has 33 exons, but the vast majority of CADASIL-related mutations are found between exons 2 and 24. NOTCH3 encodes a transmembrane receptor that contains epidermal growth factor-like domains. Accumulation and deposition of the NOTCH3 extracellular domain within vessel walls are key pathologic features of CADASIL. Most CADASIL-related NOTCH3 mutations result in the gain or loss of a cysteine residue, but others have been identified that do not include these and continue to be debated by the scientific community, including their role in CADASIL, whether they are associated with other small vessel pathologies, or even whether they have no pathogenic effect [9].

The importance of performing a cranial CT scan is not about its usefulness in diagnosing CADASIL but rather at an early stage to rule out acute hemorrhage. Brain MRI in early stages may have nonspecific findings [10], but as the disease progresses, the most common findings are white matter hyperintensities (WMHs), lacunar infarcts, and brain microhemorrhages. WMH are usually found in the anterior temporal lobe and external capsules and are typically symmetrical and bilateral. The number of lacunar infarctions is an important predictor of cognitive impairment in patients with CADASIL [7].

The NOTCH3 gene mutation identified in the clinical case, despite not having been previously described or associated with CADASIL, taking into account that both clinical manifestations and brain MRI imaging findings are in favor of this diagnosis, may correspond to a new mutation with diagnostic pathological significance in this disease.

There is still no specific and effective treatment for CADASIL. In the absence of curative approaches, treatments should be directed toward the pursuit of possible disease-modifying strategies, namely, focusing on the control of cardiovascular risk factors, particularly smoking and hypertension. The use of antiplatelet agents such as aspirin or clopidogrel in the treatment of patients with CADASIL is based on the guidelines used in sporadic stroke, however, the adequacy of this approach is undetermined due to the fact that microhemorrhages have been reported in a considerable percentage of patients with CADASIL [4].

## Conclusions

CADASIL is the most frequent monogenic disease of small cerebral vessels, whose diagnosis remains a challenge and for this, we must think of it as a diagnostic hypothesis. The diagnosis of CADASIL is based on the clinic, the findings of the brain MRI, and the confirmatory genetic test. There is still no proven effective treatment and, therefore, currently, the treatment instituted includes mainly symptomatic relief and control of risk factors. Genetic counseling should be offered to all patients diagnosed with CADASIL and their families, as early diagnosis of this disease has an important prognostic impact.
